# Phytochemical Investigations on Chemical Constituents of *Taraxacum bessarabicum* (Hornem.) Hand.-Mazz. subsp. *bessarabicum *(Hornem.) Hand.-Mazz.

**Published:** 2019

**Authors:** Aynur Sarı, Zeynep Keçeci

**Affiliations:** *Department of Pharmacognosy, Faculty of Pharmacy, Istanbul University, , Istanbul, Turkey.*

**Keywords:** Taraxacum bessarabicum subsp, bessarabicum, Asteraceae, Coumarins, Flavonoids, Phenolic acids

## Abstract

Plants of the genus *Taraxacum* Wigg., have long been used as medicinal herbs. A phytochemical investigation of the aerial parts of *Taraxacum bessarabicum* (Hornem.) Hand.-Mazz. subsp. *bessarabicum *(Hornem.) Hand.-Mazz. (Asteraceae) yielded two coumarins [esculetin (1), cichoriin (2)], three flavonoids [ luteolin (3), luteolin 7-O-β-D-glucoside (4), gossypetin (5)] and six phenolic acids and their derivatives [ p-coumaric acid (6), caffeic acid (7), ferulic acid (8), chlorogenic acid methyl ester (9), 3,5-di-O-caffeoylquinic acid (10), 3,5-di-O-caffeoylquinic acid methyl ester (11)]. Their structures were established conclusively by UV, ESI-MS, 1-D and 2-D NMR spectra analyses and comparison with literature data. The presence of these compounds has been shown for the first time from this species. This is the first report of the isolation of compound 5 from the genus *Taraxacum*.

## Introduction

The genus *Taraxacum *Wigg., commonly known as dandelion, belongs to the family Asteraceae, subfamily Cichorioideae, tribe Lactuceae (1-2). The name is derived from the Greek words ‘taraxis’, for inflammation, and ‘akeomai’ for curative (1). This plant genus has about 2500 species worldwide, and is widely distributed in the warmer temperature zones of the northern hemisphere (2-3). The total number of *Taraxacum* in Turkey at present is 43 species (4).

There are about 35.000 to 70.000 plant species that have been used for medicinal purposes worldwide (5). The plants of the genus *Taraxacum* have long been used in many traditional and modern herbal medical systems (1, 6). 

The first evidence for its therapeutic use was mentioned by Arabian physicians of the 10th and 11th centuries to treat liver and spleen ailments (1). *Taraxacum *species have been traditionally used to medicate hepatic disorders, diarrhea, viral infections, anorexia, gout, and some women’s diseases, such as breast and uterus cancers, and as lactating, choleretic, diuretic, and anti-inflammatory remedies (1, 5-6). In Turkish popular medicine, the plants of the genus *Taraxacum* are used as antirheumatic, anti-inflammatory, anti-diabetic medicines, for the treatment of eye diseases, stomach disorders, and kidney stones (9). Apart from being used as a pharmaceutical, the inflorescences, leaves and roots of *Taraxacum *species are processed into different food products. Young leaves of cultivated or wild species are consumed fresh as salad, whereas the roots are roasted and utilized as a coffee substituted. Additionally, the extracts are used as flavor components in various food products, including alcoholic beverages and soft drinks, frozen dairy desserts, candy, baked goods, gelatins and puddings, and cheese (1). 

Previous phytochemical investigations have shown that *Taraxacum *species contain sesquiterpene lactones, triterpenes, phytosterols, flavonoids, lignans, coumarins, phenolic acids, beta-carboline alkaloids, indole alkaloids, and carotenoids (1, 10-18).

*Taraxacum bessarabicum* (Hornem.) Hand.-Mazz. subsp. *bessarabicum *(Hornem.) Hand.-Mazz. is a perennial herbaceous plant. It is distributed mainly inner Anatolia and grows on saline places, fields, and 900-3000 m altitude (2). The isolation of sesquiterpene lactones and two phenolics has previously been reported from the roots of *Taraxacum bessarabicum* (11). In the present study, the aerial parts of *T. bessarabicum* subsp. *bessarabicum* collected from Erzincan, East Anatolia were investigated to elucidate their secondary-metabolite profile. 

## Experimental

*General experimental procedures*

UV Spectra: Shimadzu UV-1700 (PharmaSpec) spectrophotometer, in MeOH; λ_max_ in nm. ^1^H- and ^13^C-NMR Spectra: Varian Unity Inova 500 MHz spectrometer, at 500/125 MHz, resp., in CD_3_OD, δ in ppm rel. to Me_4_Si, J in Hz. ESIMS: Finnigan LCQ Advantage Max mass spectrometer, in m/z. Column chromatography (CC): silica gel 60 (40 – 63 μm; Merck) or Sephadex LH-20 (Sigma-Aldrich). Analitical and preparative TLC were performed on silica gel 60 F_254_ plates (0.25 and 0.50 mm, respectively; Merck). Spots were visualized by exposure to UV radiation, NH_3_ vapour, and NA spray reagent (Naturstoff reagenz-A). All solvents and chemical reagents were purchased from Merck (Darmshtot, Germany).

*Plant material*

The plant material was collected from Erzincan, East Anatolia, in August 2008. A voucher specimen (ISTE 81950) is deposited at the Herbarium of the Faculty of Pharmacy, Istanbul University, Turkey.

*Extraction and isolation*: The air-dried, ground, aerial parts of *Taraxacum bessarabicum* subsp. *bessarabicum *(730 g) were exhaustively macerated with MeOH at room temperature. After solvent evaporation, 97 g of residue was obtained, which was dissolved in MeOH/H_2_O (1:2), and then successively extracted with petroleum ether, CHCl_3_, and AcOEt. The AcOEt and CHCl_3_ layers were dried in vacuo yielding 5.18 g, 3.81 g, respectively. 

The AcOEt layer was separated by silica gel column chromatography using a step- wise gradient of CHCl_3_ and MeOH to give 34 fractions (Fr. 1-34). Fr. 8 (0.871 g) was chromatographed on a column of Sephadex LH-20 with MeOH as eluent to give 5 fractions (Fr. 8.1-8.5). Fr. 8.1 (0.272 g) was subjected to prep. TLC (silica gel; CHCl_3_ / MeOH 7:3) to provide pure 9 (6.1 mg), 6 (1.3 mg), and 2 (1.6 mg), respectively. Fr. 8.3 (0.241 g) was subjected to column chromatography (Sephadex LH-20; MeOH) and then to prep. TLC (silica gel; CHCl_3_ / MeOH 6:4) to provide pure 4 (6 mg), 11 (2.4 mg), 10 (1.3 mg), 7 (2.3 mg), and 8 (3.1 mg), respectively. Fraction 23 (0.887 g) was subjected to column chromatography (Sephadex LH-20; MeOH) and then to prep. TLC (silica gel; Toluol/AcOEt/HCOOH 5:4:1) to afford pure 5 (2.4 mg), 3 (1.1 mg), respectively. The CHCl_3 _layer was separated by silica gel column chromatography using a step- wise gradient of CHCl_3_ and MeOH to give 7 fractions (Fr. 1-7). Fr. 4 (0.887 g) was subjected to prep. TLC (silica gel; Toluol/AcOEt/HCOOH 5:4:1) and then to column chromatography (Sephadex LH-20; MeOH) to afford pure 1 (6.1 mg).

*Esculetin (1)*: White powder; UV λ_max_ (MeOH, nm): 221, 252 sh, 292, 344; ^1^H-NMR (CD_3_OD, 500 MHz): δ = 6.16 (1H, d, J = 9.3 Hz, H-3), 6.73 (1H, s, H-8), 7.08 (1H, s, H-5), 7.85 (1H, d, J = 9.3 Hz, H-4). 

*Cichoriin (2)*: White powder; UV λ_max_ (MeOH, nm): 226, 251, 287, 340; ^1^H-NMR (CD_3_OD, 500 MHz): δ = 3.42-3.55 (4H, m, H-2′-5′), 3.72 (1H, dd, J = 2.0, 12.2 Hz, H-6′a), 3.93 (1H, dd, J = 5.8, 12.2 Hz, H-6′b), 4.98 (1H, d, J = 7.3 Hz, H-1′), 6.29 (1H, d, J = 9.3 Hz, H-3), 7.05 (1H, s, H-8), 7.21 (1H, s, H-5), 7.83 (1H, d, J = 9.3 Hz, H-4). 

*Luteolin* *(3)*: Pale yellow crystals; UV λ_max_ (nm): (MeOH) 251, 268 sh, 348 nm, (MeOH+NaOMe) 273, 323 sh, 406 401, (MeOH+AlCl_3_) 275, 298 sh, 428, (MeOH+AlCl_3_+HCl) 265, 276 sh, 370, (MeOH+NaOAc) 274, 328, 362, (MeOH+NaOAc+H_3_BO_3_) 262, 380; ^1^H-NMR (acetone-d_6_, 500 MHz): δ = 6.32(1H, d, J = 1.9 Hz, H-6), 6.51 (1H, d, J = 1.9 Hz, H-8), 6.61 (1H, s, H-3), 6.92 (1H, d, J = 8.4 Hz, H-5′), 7.43 (1H, dd, J = 1.9, 8.4 Hz, H-6′), 7.48 (1H, d, J = 1.9 Hz, H-2′). 

*Luteolin 7-O-β-D-glucoside (4)*: Pale yellow crystals; UV λ_max_ (nm): (MeOH) 254, 268 sh, 342, (MeOH+NaOMe) 267, 401, (MeOH+AlCl_3_) 274, 298 sh, 429, (MeOH+AlCl_3_+HCl) 276, 387, (MeOH+NaOAc) 267, 381, (MeOH+NaOAc+H_3_BO_3_) 261, 370; ^1^H-NMR (CD_3_OD, 500 MHz): δ = 3.40-3.70 (4H, m, H-2″-5″), 4.21 (1H, dd, J = 6.8, 11.7 Hz, H-6″a), 4.44 (1H, dd, J = 2.0, 11.7 Hz, H-6″b), 4.97 (1H, d, J = 7.3 Hz, H-1″), 6.41 (1H, d, J = 1.9 Hz, H-6), 6.51 (1H, s, H-3), 6.67 (1H, d, J = 1.9 Hz, H-8), 6.82 (1H, d, J = 8.4 Hz, H-5′), 7.30 (1H, d, J = 1.9 Hz, H-2′), 7.33 (1H, dd, J = 1.9, 8.4 Hz, H-6′).

*Gossypetin (5)*: Yellow powder; UV λ_max_ (MeOH, nm): 285, 379, (MeOH+NaOMe) 283, 414, (MeOH+AlCl_3_) 279, 432, (MeOH+AlCl_3_+HCl) 283, 392, (MeOH+NaOAc) 282, 407, (MeOH+NaOAc+H_3_BO_3_) 283, 399; ESI-MS (negative): m/z 353.4 [ M+Cl]^-^; ^1^H-NMR (CD_3_OD, 500 MHz): δ = 6.46 (1H, s, H-6), 6.90 (1H, d, J = 8.3 Hz, H-5′), 7.43 (1H, dd, J = 1.9, 8.3 Hz, H-6′), 7.48 (1H, d, J = 1.9 Hz, H-2′). 

*p-Coumaric acid (6*): White powder; UV λ_max_ (MeOH, nm): 214, 226, 301, 310; ^1^H-NMR (CD_3_OD, 500 MHz): δ = 6.42 (1H, d, J=15.7 Hz, H-8), 6.79 (2H, d, J=7.32 Hz, H-3 and H-5), 7.39 (2H, d, J = 7.32 Hz, H-2 and H-6), 7.48 (1H, d, J = 15.7 Hz, H-7).

*Caffeic acid (7)*: White powder; UV λ_max_ (MeOH, nm): 216, 242, 295sh, 324; ^1^H-NMR (CD_3_OD, 500 MHz): δ = 6.42 (1H, d, J = 16.1 Hz, H-8), 6.77 ( 1H, d, J = 7.8 Hz, H-5), 6.95 (1H, dd, J = 1.9, 7.8 Hz, H-6), 7.07 (1H, d, J = 1.9 Hz, H-2), 7.62 (1H, d, J = 16.1 Hz, H-7); ^13^C-NMR (CD_3_OD, 100 MHz, signal assignment by HSQC and HMBC experiments): 114.1 (C-2), 115.0 (C-8), 115.2 (C-5), 121.7 (C-6), 127.2 (C-1), 145.5 (C-3), 145.7 (C-7), 148.5 (C-4), 167.8 (C-9).

*Ferulic acid (8)*: White powder; UV λ_max_ (MeOH, nm): 218, 232, 293sh, 321; ^1^H-NMR (CD_3_OD, 500 MHz): δ= 3.89 (3H, s, OMe), 6.50 (1H, d, J = 15.6 Hz, H-8), 6.80 ( 1H, d, J = 8.3 Hz, H-5), 7.09 (1H, dd, J = 1.9, 8.3 Hz, H-6), 7.21 (1H, brs, H-2), 7.69 (1H, d, J = 15.6 Hz, H-7); ^13^C-NMR (CD_3_OD, 100 MHz, signal assignment by HSQC and HMBC experiments): 55.2 (OMe), 110.5 (C-2), 115.0 (C-8), 115.2 (C-5), 122.8 (C-6), 127.2 (C-1), 148.5 (C-3), 145.0 (C-7), 149.0 (C-4), 167.8 (C-9).

*Chlorogenic acid methyl ester (9)*: White powder; UV λ_max_ (MeOH, nm): 218, 232, 243 sh, 299 sh, 328; ^1^H-NMR (CD_3_OD, 500 MHz): δ = 2.01 (1H, dd, J = 6.3, 13.6 Hz, H-2a), 2.16 (2H, m, H-6), 2.21 (1H, dd, J = 3.0, 13.6 Hz, H-2b), 3.63 (3H, s, OMe), 3.73 (1H, dd, J = 3.4, 13.6 Hz, H-4), 4.13 and 5.28 (1H each, m, H-3 and H-5), 6.22 (1H, d, J = 16.1 Hz, H-8′), 6.78 (1H, dd, J = 1.9, 8.3 Hz, H-6′), 6.95 ( 1H, d, J=8.3 Hz, H-5′), 7.04 (1H, d, J = 1.9 Hz, H-2′), 7.53 (1H, d, J = 16.1 Hz, H-7′).

*3,5-Di-O-caffeoylquinic acid (10)*: White powder; UV λ_max_ (MeOH, nm): 218, 235, 244, 299 sh, 328; ^1^H-NMR (CD_3_OD, 500 MHz): δ= 1.94 (3H, m, H-2a and H-6), 2.10 (1H, dd, J = 7.3, 14.2 Hz, H-2b), 4.10 (1H, dd, J = 3.0, 7.8 Hz, H-4), 5.26 (2H, m, H-3 and H-5), 6.28 and 6.30 (1H each, d, J = 15.6 Hz, H-8′, -8″), 6.68 and 6.69 ( 1H each, d, J = 8.3 Hz, H-5′, -5″), 6.86 and 6.89 (1H each, dd, J = 1.9, 8.3 Hz, H-6′, -6″), 6.98 and 6.99 ( 1H each, d, J = 1.9 Hz, H-2′, -2″), 7.51 and 7.58 ( 1H each, d, J = 15.6 Hz, H-7′, -7″); ^13^C-NMR (CD_3_OD, 100 MHz, signal assignment by HSQC and HMBC experiments): δ = 36.5 (C-2), 37.4 (C-6), 70.9 (C-4), 72,3 (C-3), 72,7 (C-5), 74.6 (C-1), 115.2 (C-8′, C-8′′ ), 115.7 and 115.8 (C-2′, C-2′′ ), 116.8 (C-5′, C-5′′ ), 123.1 and 123.2 (C-6′, C-6′′ ), 128.0 (C-1′, C-1′′ ), 145.8 (C-3′, C-3′′ ), 146.9 and 147.1 (C-7′, C-7′′ ), 148.9 and 149.3 (C-4′, C-4′′ ), 168.6 (C-9′, C-9′′ ), 175.4 (C-7). 

**Figure 1:**
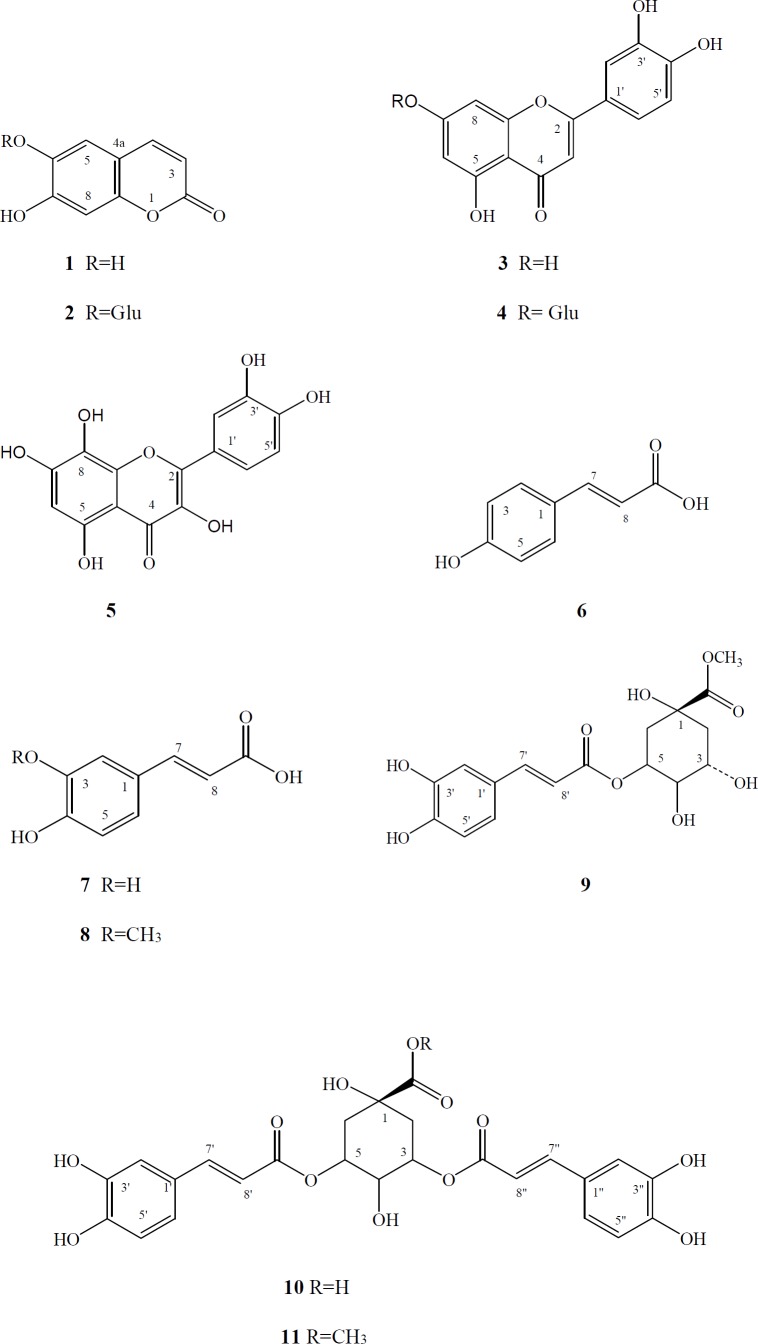
Structures of compounds **1-11** isolated from *Taraxacum bessarabicum *subsp. *bessarabicum*

*3,5-Di-O-caffeoylquinic acid methyl ester (11)*: White powder; UV λ_max_ (MeOH, nm): 219, 233 sh, 244, 299 sh, 331; ESI-MS (negative): m/z 529.15 [M-1]^- ^; ^1^H-NMR (CD_3_OD, 500 MHz): δ = 2.15 (1H, dd, J = 6.3, 14.2 Hz, H-2a), 2.20 (2H, m, H-6), 2.31 (1H, dd, J = 3.4, 14.2 Hz, H-2b), 3.53 (3H, s, OMe), 3.98 (1H, dd, J = 2.9, 7.8 Hz, H-4), 5.32 (1H, m, H-5), 5.40 (1H, m, H-3), 6.22 and 6.34 (1H each, d, J = 15.6 Hz, H-8′, -8″), 6.78 and 6.79 ( 1H each, d, J = 8.3 Hz, H-5′, -5″), 6.97 (2H, brd, J = 8.3 Hz, H-6′, -6″), 7.06 and 7.07 ( 1H each, d, J = 1.9 Hz, H-2′, -2″), 7.55 and 7.62 ( 1H each, d, J = 15.6 Hz, H-7′, -7″). 

## Results and Discussion

Dietary phytochemicals constitute a relevant research area of nutrition and health. The future of this area depends on the identification of active molecules within foods and plants and on an increased understanding of how the use of such molecules might play a role in disease prevention and therapy (25).

Dandelion (*Taraxacum* species) contains a wide array of phytochemicals whose biological activities are actively being explored in various areas of human health. In particular, emerging evidence suggests that dandelion and its constituents have antioxidant and anti-inflammatory activities that result in diverse biological effects (1). Bitter substances are known for their stimulation of the digestion, while phenolic compounds are accounted for the anti-inflammatory and antioxidative activity of plant extracts. Therefore, focus was set on the elucidation of such pharmacologically important compounds in dandelion plants in the past decades (1, 3).

The therapeutic actions of *Taraxacum *species have partially been ascribed to their bitter principles, more precisely to some sesquiterpenes. Other constituents isolated from dandelion include various triterpenes and phytosterols, phenolic compounds, and sugars, among others, found in the organs of the plant (1). 

To wit, in spite of all the researches carried out, less than 1% of all the species identified so far (over 2500) have been studied (including *Taraxacum officinale*,* Taraxacum coreanum*,* Taraxacum laevigatum*,* Taraxacum mongolicum and Taraxacum platycarpum*). This is an indication of the little knowledge that we have about this genus so far (3). 

*Taraxacum bessarabicum* (Hornem.) Hand.-Mazz. subsp. *bessarabicum *(Hornem.) Hand.-Mazz. is a perennial herbaceous plant. The isolation of sesquiterpene lactones and two phenolics has previously been reported from the roots of *Taraxacum bessarabicum* (11). Compared to roots, dandelion aerial parts are characterized by higher polyphenol contents (1). In the current study, the aerial parts of *T. bessarabicum* subsp. *bessarabicum* collected from Erzincan, East Anatolia were investigated to elucidate their secondary-metabolite profile. Two coumarins [esculetin (1), cichoriin (2)], three flavonoids [ luteolin (3), luteolin 7-O-β-D-glucoside (4), gossypetin (5)] and six phenolic acids and their derivatives [ p-coumaric acid (6), caffeic acid (7), ferulic acid (8), chlorogenic acid methyl ester (9), 3,5-di-O-caffeoylquinic acid (10), 3,5-di-O-caffeoylquinic acid methyl ester (11)] (Figure. 1) have been isolated from the EtOAc and CHCl_3_ fractions of the MeOH extract from the aerial parts of *Taraxacum bessarabicum* (Hornem.) Hand.-Mazz. subsp. *bessarabicum *(Hornem.). Column chromatography and preparative thin layer chromatography were used for separation of these compounds. Their structures were established conclusively by UV, ESI-MS, 1-D and 2-D NMR spectra analyses and comparison with literature data (13, 16-24). The results demonstrate that this is the first report of the isolated compounds from T. *bessarabicum *subsp. *bessarabicum*. Compound 5 is new for the genus *Taraxacum*. 

This represents the first record in the genus *Taraxacum* of flavonol with extra 8-hydroxyl substituent.
